# An integrated view of sex differences in metabolic physiology and disease

**DOI:** 10.1016/j.molmet.2018.06.011

**Published:** 2018-06-21

**Authors:** Deborah J. Clegg, Franck Mauvais-Jarvis

**Affiliations:** 1 Division of General Internal Medicine and Department of Biomedical Sciences Cedars-Sinai Medical Center, Los Angeles, CA, USA; 2 Diabetes Discovery Research and Gender Medicine Laboratory, Department of Medicine, Section of Endocrinology and Metabolism, Tulane University Health Sciences Center, School of Medicine, and Southeast Louisiana Veterans Healthcare System Medical Center, New Orleans, LA 70112, USA

Due to the fundamental biological differences between males and females, nearly all aspects of metabolism, including energy balance as well as glucose and lipid metabolism, are regulated in a sexually dimorphic manner and influence the pathogenesis of most, if not all, diseases, including obesity and diabetes [1]. This special issue contains a series of reviews demonstrating the importance of understanding mechanisms which underlie sex differences in metabolic function and their implications for diabetes and obesity. Additionally, articles highlight methods used in beginning to address how and when sex-based differences occur and how to test for them.

Maggi et al. propose the paradigm that females were exposed to significantly higher evolutionary pressure than males that perfected the coupling between energy metabolism and reproduction. This is exemplified by neonatal liver sexual differentiation and the concept that liver sexual dimorphism is associated with female reproductive functions.

All biological sex differences originate with the differences in sex chromosomes [2], [3]. To address this issue, Zore et al. discuss sex differences in adiposity and obesity, focusing on the influence of XX chromosome dosage on sex differences in adiposity and the dosage effects of sex chromosomes on lipid metabolism and levels.

Early life programming by the testicular testosterone surge in males is the second most important factor in wiring sex differences that are observed in the adult [2], [3]. The early life environments of a fetus in utero and during the neonatal period are major determinants in programming sex differences in physiology and later life susceptibility to cardio-metabolic disease. Dearden et al. dissect the developmental programming of sexual dimorphisms in systems regulating energy homeostasis and the role of early life adverse conditions such as undernutrition and obesity in programing diseases. In addition to sex chromosomes and the testicular testosterone surge, the gonadal hormone surge seen during puberty is an additional factor, which activates an additional level of sexual dimorphisms [2], [3]. In that perspective, Palmisato et al. explore the effect of estrogens in driving sex differences in lipid and lipoprotein metabolism and how they influence metabolic and cardiovascular diseases. Cross et al. summarize the current knowledge associated with the complex interplay between sexual dimorphisms in cardiometabolic disease and the gut microbiome with the concept that gut microbiota maintains sex hormone levels and influences reproduction. Gannon et al. focus on clinical and experimental sex differences in islet cell biology and dysfunction, discussing the concept of ovarian- and testicular–islet axes in the sex-specific fine-tuning of insulin secretion and the influence sex hormones play on diabetes. Most metabolic effects of estrogens are mediated via estrogen receptor-α (ERα) [4], one of the estrogen receptors. Xu and Lopez explore the role of the estrogen-ERα axis in the central nervous system regulation of energy balance while Zhou et al. focus on the estrogens-ERα interaction and their influence on skeletal muscle insulin sensitivity. The subcellular localization of estrogen receptors and their communication between membrane, cytoplasmic and nuclear pools is a critical aspect of ER signaling. Guillaume et al. dissect the influence of the location and activity of ERα on cardiometabolism. Importantly, estrogens and androgens do not just act as circulating endocrine hormones. They can also be interconverted as discussed by Katya Rubinow, who explores the role of local sex steroid synthesis and production by immune cells and the relevance of immune cell intracrinology to sex differences in metabolic disease.

The role of sex is a fundamental issue in medicine. As is highlighted by these reviews in this special issue of *Molecular Metabolism*, the combined actions of sex chromosomes and sex hormones throughout life influence nearly all aspects of metabolic function. Sex has long been ‘ignored’ in science, and male animals have been predominately used in basic research due to the ‘complexity’ of studying females because of their naturally fluctuating sex hormones. As we have come to learn, males and females are biologically different, and both sexes need to be included in all facets of research from the cell to the whole organism. The over-reliance on male animals and cells in preclinical research blinds discovery of sex differences that could guide studies to prevent metabolic disease. In fact, there is an economic advantage in considering sex early in basic research. The generation of sex-specific information may enhance the design of clinical trials to promote the development of relevant sex-based treatment for obesity and diabetes.

## Conflict of interest

The authors declare no conflict of interest.
